# Thermal assisted up-conversion electroluminescence in quantum dot light emitting diodes

**DOI:** 10.1038/s41467-022-28037-w

**Published:** 2022-01-18

**Authors:** Qiang Su, Shuming Chen

**Affiliations:** 1 Department of Electrical and Electronic Engineering, Southern University of Science and Technology, Shenzhen, 518055 People’s Republic of China; 2 Key Laboratory of Energy Conversion and Storage Technologies (Southern University of Science and Technology), Ministry of Education, Shenzhen, 518055 People’s Republic of China

**Keywords:** Electronic devices, Inorganic LEDs

## Abstract

Up-conversion electroluminescence, in which the energy of a emitted photon is higher than that of the excitation electron, is observed in quantum-dot light-emitting diodes. Here, we study its mechanism by investigating the effect of thermal energy on the charge injection dynamic. Based on the results of temperature-dependent electroluminescence and theoretical analysis, we reveal that at sub-bandgap voltage, holes can be successfully injected into quantum-dots via thermal-assisted thermionic-emission mechanism, thereby enabling the sub-bandgap turn-on and up-conversion electroluminescence of the devices. Further theoretical deduction and experimental results confirm that thermal-assisted hole-injection is the universal mechanism responsible for the up-conversion electroluminescence. This work uncovers the charge injection process and unlocks the sub-bandgap turn-on mechanism, which paves the road for the development of up-conversion devices with power conversion efficiency over 100%.

## Abstract (web summary)

How holes are injected into quantum dots at sub-bandgap bias has puzzled researchers for years. Here, authors show, using temperature-dependent electroluminescence (EL) and theoretical analysis, such injection occurs via thermal-assisted thermionic-emission, consequently enabling the sub-bandgap turn-on and up-conversion EL.

## Introduction

The past decade has witnessed the fast development of quantum-dot (QD) light-emitting diodes (QLEDs), which are considered to be the ideal candidates for next generation displays because they are self-emitting and have the advantages of high color saturation, high efficiency and low cost processibility^1–8^. Typical QLEDs consist of a p-type polymeric hole transport layer (HTL), a QD emitter and a n-type ZnO electron transport layer (ETL), which are sequentially stacked and sandwiched between a transparent anode and a metallic cathode^1–10^. By applying a voltage, electrons and holes can overcome the potential barrier and inject into the QDs, subsequently forming the electron-hole pairs (excitons) that are bound by their Coulombic attraction. The radiative recombination of excitons leads to the generation of photons with energy of $h\nu$, which is roughly equal to or slightly smaller than the bandgap energy (*E*_*g*_) of the QDs. Because the photons are converted from the electrons, the energy of the injected electrons at a applied voltage of *V* should thus be equal to the energy of photons, i.e., ${eV}=h\nu \approx {E}_{g}$, which implies that the minimum applied voltage or the turn-on voltage ${V}_{T}$ to induce detectable electroluminescence (EL) should satisfy ${V}_{T}\ge h\nu /e$. However, it has been frequently observed that the ${V}_{T}$ is substantially smaller than $h\nu /e{{{{{\rm{;}}}}}}$^1,3–5,11^ for instance, we found that the turn-on voltage to induce a 620 nm red emission in typical QLEDs can be as low as 1.2 V (Supplementary movie), which is far smaller than the bandgap voltage of 2 V and means that an electron with energy of 1.2 eV can be up-converted to a 2.0 eV photon. The phenomena of sub-bandgap turn-on and up-conversion EL have also been observed in III-V compound semiconductor LED^12–14^, Rubrene/Fullerene organic LED^15–17^ and MEH-PPV/ZnO LED^18^, but the mechanism remains unclear and is still under debate.

For typical QLEDs driven by a sub-bandgap voltage, electron injection into QDs is relatively efficient, but hole injection into QDs is extremely difficult because of the presence of the heterojunction barrier, and thus most holes have to accumulate at the hetero-interface of QDs/HTL^19^. Therefore, unveiling the hole injection process is the key to unlock the mechanism of up-conversion EL. Most reports conclude that hole injection into QDs is enabled by an Auger-assisted process^1,2,4,20^, in which the interfacial excitons formed between the injected electrons and the accumulated holes resonantly transfer their energy to the proximal holes, thereby providing extra energy to the holes and assisting them to overcome the barrier and inject into the QDs. In such a process, the formation of one QD exciton should consume an interfacial exciton, and thus the maximum internal quantum efficiency (IQE) is limited to 50% and thus the maximum external quantum efficiency (EQE) is lower than 10% by assuming a typical outcupling efficiency of 20~25%^6^. However, most efficient QLEDs can exhibit a high EQE of over 10% even driven by a sub-bandgap voltage^3,5,21^, thereby disapproving the Auger-assisted mechanism. Another negative evidence is the turn-on voltage to induce a detectable EL is smaller than that required to create the interfacial excitons^22^. Therefore, the Auger process is unlikely responsible for the up-conversion EL. Recently, Chen et al. revealed that the turn-on voltage of red QLEDs is approximately equal to the flat-band (FB) voltage of the QD layer and they concluded that when the QD layer reaches the FB state, holes are able to inject into the QDs via the field-assisted thermionic-emission mechanism^22^, which seems reasonable to explain the up-conversion EL. Very recently, Jin et al. deciphered the exciton-generation processes in QLEDs and suggested that hole injection is assisted by confinement-enhanced Coulomb interactions^23^, which enables the devices to exhibit efficient EL at sub-bandgap voltage.

Considering that the mechanism for the up-conversion EL is still under debate, it is of fundamental interest to find out the true origin. Here, we address a fundamental question of how the holes are injected into the QDs at sub-bandgap bias. By investigating the temperature-dependent EL including current–voltage–luminance (J–V–L), capacitance–voltage (C–V) and transient EL characteristics, we are able to probe the effect of thermal energy on the charge injection dynamic. We reveal that thermal energy play an essential role in the sub-bandgap charge injection processes. At sub-bandgap bias, holes can be successfully injected into QDs via thermal-assisted thermionic-emission mechanism, thereby enabling the sub-bandgap turn-on and up-conversion EL of the devices. Our findings uncover the charge injection process and unlock the sub-bandgap turn-on mechanism, which could encourage the development of up-conversion QLEDs with power conversion efficiency over 100%.

## Results

### Thermal-assisted up-conversion EL

**Fig. 1:**
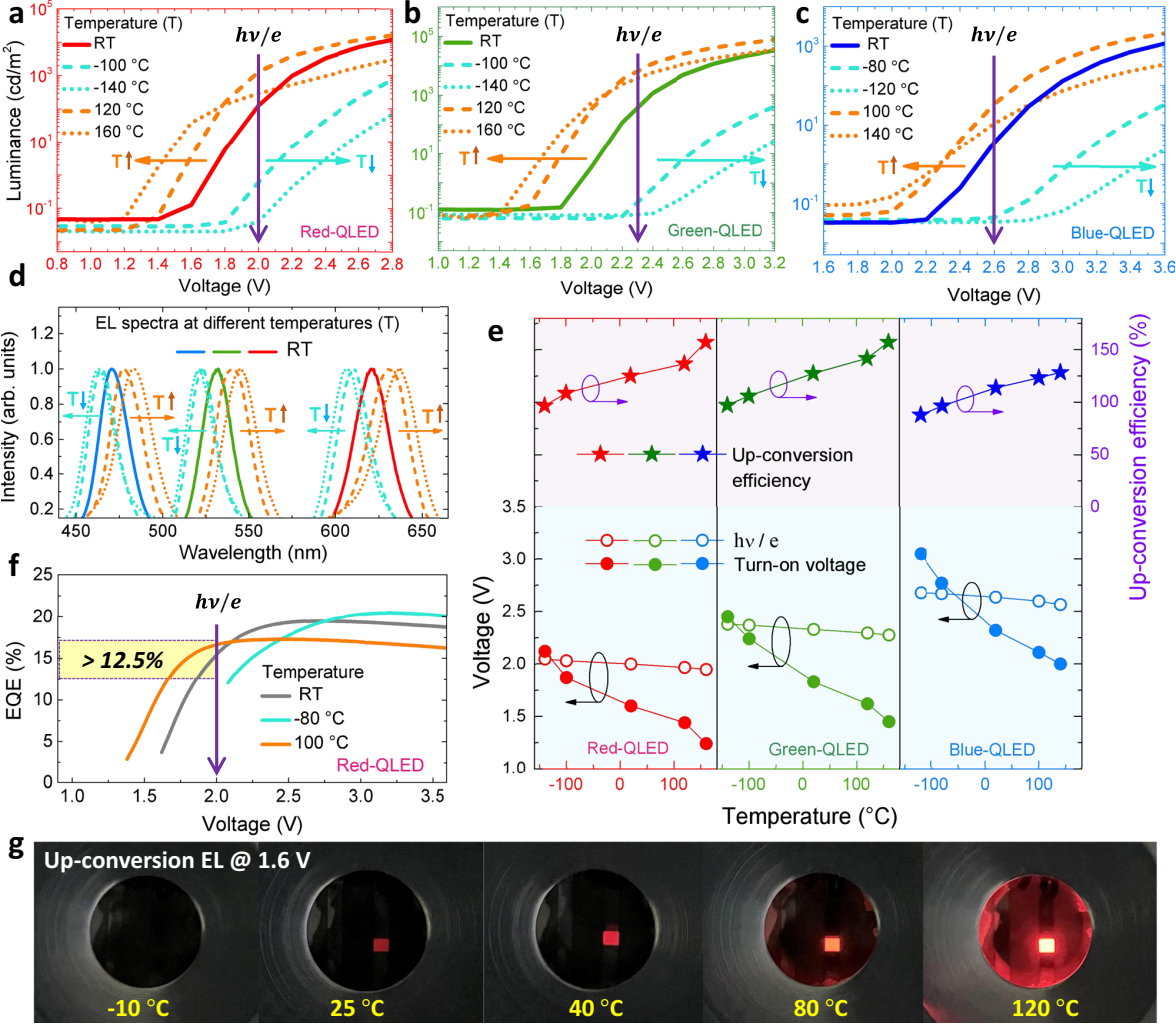
Temperature-dependent EL characteristics. The luminance-voltage (L–V) characteristics of **a** red-, **b** green-, and **c** blue-QLEDs under different temperatures (RT= room temperature): the *V*_*T*_ (turn-on voltage) is remarkably reduced as the temperature is increased. **d** EL spectra of red-, green- and blue-QLEDs at different temperatures (the orange/blue arrows represent an increase/decrease in temperature). **e** The *V*_*T*_ (solid circle), photon voltage *V*_*ph*_ (${V}_{{ph}}=h\nu /e$, open circle) and up-conversion efficiency (star) of red-, green- and blue-QLEDs at different temperatures: the up-conversion efficiency is gradually increased as the temperature is increased, indicating that the up-conversion EL is triggered by a thermal-assisted process. **f** EQE-V characteristics of red-QLEDs at different temperatures: at sub-bandgap bias of 1.7~2.0 V, the devices exhibit an EQE of 12.5~15%, which is higher than the upper limit (~10%) of the Auger-assisted process, thus disapproving the Auger-assisted mechanism. **g** Photographs of up-conversion EL at 1.6 V under different temperatures: the luminance is gradually increased as the temperature is elevated (also demonstrated in Supplementary Movie 1).

The effect of thermal energy on the EL characteristics of the QLEDs (with a regular structure of glass/ITO/PEDOT: PSS/TFB/QD/ZnMgO/Al) was investigated by varying the temperature during measurement. Fig 1a–c show the temperature-dependent luminance-voltage characteristics of the red, the green and the blue QLEDs, respectively. See Supplementary Figs. 1 and 2 for relevant performance and detailed disscussion. At room temperature (RT), all devices exhibit sub-bandgap turn-on characteristics; for instance, the turn-on voltages (${V}_{T}$) to induce a luminance of 0.1 cd m^−2^ of red (620 nm), green (532 nm) and blue (470 nm) emission are 1.6, 1.8 and 2.3 V, respectively, which are remarkably lower than their corresponding photon voltages (${V}_{{ph}}=h\nu /e$) of 2.0, 2.3 and 2.6 V, consequently resulting in the up-conversion efficiencies (${V}_{{ph}}/{V}_{T}$) or gains of 125%, 128% and 113%, respectively. Interestingly, the ${V}_{T}$ is significantly affected by the temperature; for example, for the red QLEDs, the ${V}_{T}$ is decreased to 1.25 V at an elevated temperature of 160 °C, while it is increased and approached to the bandgap voltage of 2.03 V by cooling down the devices to −100 °C. It should be noted that, even at a sub-bandgap applied voltage, the EL spectra are identical to those at high voltage (Supplementary Fig. 3), indicating that the emission is originated from the QDs. At a sub-bandgap bias of 1.6 V, the up-conversion EL is switched on at RT and is gradually enhanced when the temperature is further elevated, as demonstrated in Fig. 1g and Supplementary Movie 1. Similar phenomena are also observed in green and blue QLEDs. As shown in Fig. 1d, the EL spectra are also modulated by the temperature, which are red-shifted when the temperature is increased. This is because temperature can induce the expansion of the crystal lattice, thereby leading to the shrinkage of the QD bandgap, as well-explained by the Varshni relation^24^. To investigate whether the variation of ${V}_{T}$ is caused by the change of the photon energy, we compare the ${V}_{T}$ and ${V}_{{ph}}$ at different temperatures. As shown in Fig. 1e, for the red QLEDs, when the temperature is increased, the ${V}_{T}$ is significantly reduced (from 2.12 to 1.25 V), while the ${V}_{{ph}}$ is mildly decreased (from 2.04 to 1.95 V), and as a result, the up-conversion efficiency is rapidly increased from 96% to 156%. These results indicate that: (1) the reduction of ${V}_{T}$ at elevated temperatures should not be attributed to the change of ${V}_{{ph}}$, but instead, should be ascribed to the increase of the thermal energy; (2) the sub-bandgap turn-on and up-conversion EL are enabled by the thermal-assisted charge injection process. It is likely that thermal energy can effectively promote the charge injection, as will be discussed later. With higher thermal energy provided, more charges can be injected into QDs, thereby leading to the reduction of ${V}_{T}$ and the improvement of up-conversion efficiency, as reflected in Fig. 1e. It should be noted that the Auger-assisted charge injection process is excluded, because the devices exhibit an EQE of 12.5%~15% at a sub-bandgap bias of 1.7–2.0 V (Fig. 1f), which is higher than the upper limit of ~10% of the Auger process. At elevated temperatures, although the charge injection is promoted (Supplementary Fig. 1) and the brightness is enhanced, the EQE is reduced (Fig. 1f) due to the damage of QDs and the quenching of excitons. As shown in Supplementary Fig. 2, at elevated temperatures, the electrons are delocalized to the surface traps first and then relax back to the core and recombine with the confined holes^24^, thereby leading to the prolonged exciton lifetime (Supplementary Fig. 2b). In such a thermal-assisted recombination process^24^, the quenching possibility is increased and thus the photoluminescence (PL) intensity of QDs is reduced (Supplementary Fig. 2a).

### Charge-injection processes in QLEDs

**Fig. 2:**
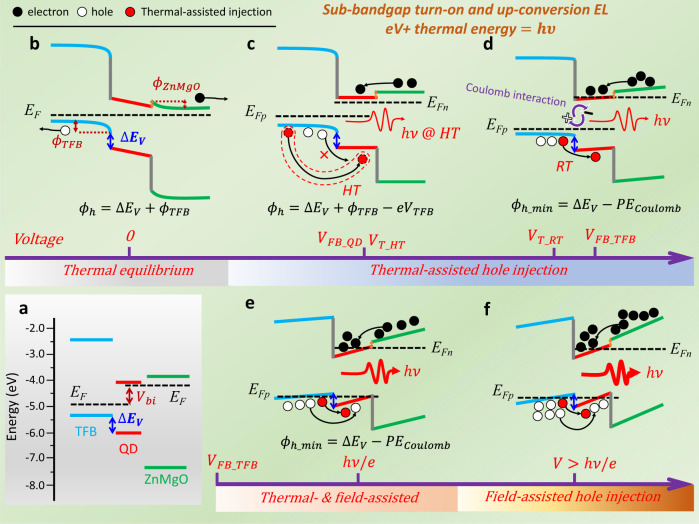
Charge injection processes in QLEDs. **a** Energy levels of the functional layers of the typical red-QLEDs. **b** At thermal equilibrium, the surfaces of TFB and ZnMgO are depleted so that the Fermi levels are aligned through the system. Due to the presence of build-in surface potentials (${\upphi }_{{{{{{\rm{TFB}}}}}}}$ and ${\upphi }_{{{{{{\rm{ZnMgO}}}}}}}$), charge injection is impossible. **c** At ${V}_{{FB\_QD}}$, flat-band is achieved in QD layer and thus electron injection into QDs is possible, while hole injection is still unfavorable due to the large injection barrier ${\phi }_{h}$. However, at high temperature (HT), with sufficient thermal energy provided, holes could be injected into QDs via the thermal-assisted thermionic-emission mechanism. **d** At ${V}_{{FB\_TFB}}$, flat-band is achieved in TFB layer, and the hole injection barrier is reduced to a minimum value of ${\phi }_{h}=\triangle {E}_{V}-{{PE}}_{{Coulomb}}$. At RT, with the assistance of thermal energy, the holes can overcome a barrier of 0.4 eV and injected into QDs. **e** At $h\upsilon /e$, all depletion regions are vanished and the electric field in all layers turn positive, and thus the holes can be accelerated towards the QDs. Hole injection is enabled by both thermal- and field-assisted thermionic-emission mechanisms. **f** At $V \, > \, h\nu /e$, due to the presence of strong positive electric field in TFB, hole injection is mainly dominated by the field-assisted thermionic-emission mechanism.

To understand how the charges are injected into QDs at sub-bandgap bias, the charge injection processes are analyzed. Fig 2a shows the energy levels of each functional layer of the red QLEDs. For simplicity, we assume ohmic contacts are achieved so that hole injection from anode into TFB and electron injection from cathode into ZnMgO are very efficient. The electrons can be readily injected from ZnMgO into QDs due to the negligible barrier of QDs/ZnMgO heterojunction, while hole injection from TFB into QDs is extremely difficult as a high heterojunction barrier ($\triangle {E}_{V}$) of:

1 $$\triangle {E}_{V}={E}_{{V}_{\_}{{QD}}}-{E}_{{HOM}{O}_{\_}{{TFB}}}$$

is presented at the TFB/QDs interface, where ${E}_{{V\_QD}}\,$and ${E}_{{HOMO\_TFB}}$ are the valance band levels of QDs and TFB, respectively. Under different applied voltages, the charge injection process is analyzed as below:

(1) $V=0{V}$. At thermal equilibrium, the surfaces of TFB and ZnMgO are depleted so that the Fermi levels are aligned through the system, as displayed in Fig. 2b. Because of the existence of surface depletion layer, build-in surface potentials ${\phi }_{{TFB}}$ and ${\phi }_{{ZnMgO}}$ are created at the surface of TFB and ZnMgO, respectively. In this situation, injection of holes and electrons is impossible because they will be pulled back by the negative build-in potentials.

(2)$\,0 \, < \, V\le {V}_{{FB\_QD}}$ (${V}_{{FB\_QD}}$ defined as the flat-band voltage of QDs). The applied voltage, which is in opposite direction to the build-in potentials, is mainly dropped across the depletion layers. As shown in Fig. 2c, when the applied voltage is increased to ${V}_{{FB\_QD}}$, corresponding to the flat-band voltage of QDs, electron injection into QDs becomes possible, while hole injection from the depleted TFB into QDs is still unfavorable due to the presence of injection barrier. The hole injection barrier is determined by:

2 $${\phi }_{h}=\triangle {E}_{V}+{\phi }_{{TFB}}-e{V}_{T{FB}}$$

where ${V}_{{TFB}}$ is the effective applied voltage that is dropped across the depletion region of TFB. The accumulated electrons in QDs tend to attract the holes in TFB via the Coulombic interaction^23,25^. The potential energy of holes induced by the Coulombic interaction (${{PE}}_{{Coulomb}}$) is:

3 $${{PE}}_{{Coulomb}}=\frac{{e}^{2}}{4\pi {\varepsilon }_{0}{\varepsilon }_{r}x}$$

where *x* is the distance between electrons and holes, and ${\varepsilon }_{0}$, ${\varepsilon }_{r}$ are the vacuum dielectric permittivity and the relative dielectric constant of TFB, respectively. Considering the Coulombic interaction, the injection barrier then is modified as (also shown in Supplementary Figure 4):

4 $${\phi }_{h}=\triangle {E}_{V}+{\phi }_{{TFB}}-e{V}_{{TFB}}-{{PE}}_{{Coulomb}}$$

It should be noted that when the surface of TFB is depleted, the ${{PE}}_{{Coulomb}}$ could be neglected because electrons and holes are separated by a long distance. If the kinetic energy of holes (${E}_{K}$), gained from thermal energy, is larger than ${\phi }_{h}$, then the holes can be injected into QDs via the thermionic-emission mechanism^14,25^. At RT and ${V}_{{FB\_QD}}$, it is difficult to observe the EL; however, at high temperature (HT), with sufficient thermal energy provided, thermal-assisted hole injection could be triggered, thereby enabling the turn-on of EL at ${V}_{{FB\_QD}}$. It should be noted that the ${V}_{{FB\_QD}}$ is the minimum voltage to initiate the EL^22^, and thus by measuring the lowest ${V}_{T}$, the ${V}_{{FB\_QD}}$ can be accessed. As shown in Supplementary Fig. 5, when the temperature is increased from 160 to 240 °C, the ${V}_{{T\_HT}}$ (turn-on voltage at HT) cannot be further reduced and is fixed at ~1.2 V, which represents the lowest ${V}_{T}$ and therefore marks the value of ${V}_{{FB\_QD}}$. A general picture to describe the up-conversion EL is that, at ${V}_{{FB\_QD}}$, an electron with energy of 1.2 eV can be directly injected into QDs, while with the assistance of thermal energy, a hole can overcome the ${\phi }_{h}$ and inject into QDs, thereby producing a 2.0 eV photon with a up-conversion gain of 167%. Based on the energy conservation law, the hole injection barrier of 0.8 eV can readily be deduced by subtracting the electron electrostatic energy from the photon energy.

(3) ${V}_{{FB\_QD}} \, < \, V\le {V}_{{FB}{{{{{\rm{\_}}}}}}{TFB}}$ (Fig. 2d, ${V}_{{FB\_TFB}}$ defined as the flat-band voltage of TFB). As the ${V}_{{TFB}}$ dropped across the depleted TFB is increased, the ${\phi }_{h}$ is gradually reduced. For example, when the applied voltage is increased to 1.6 V, we can detect the 2.0 eV photons at RT, indicating that the ${\phi }_{h}$ is reduced to 0.4 eV. When the applied voltage is further increased to ${V}_{{FB}{{{{{\rm{\_}}}}}}{TFB}}$, corresponding to the flat-band voltage of TFB, the build-in ${\phi }_{{TFB}}$ is completely canceled, and thus the ${\phi }_{h}$ is reduced to a minimum value of:

5 $${\phi }_{h{\_}{\min }}=\triangle {E}_{V}-{{PE}}_{{Coulomb}}$$

At ${V}_{{FB}{\_}{TFB}}$, holes accumulation at the TFB/QD interface becomes possible and thus the ${{PE}}_{{Coulomb}}$ cannot be neglected, which is estimated to be ~0.1 eV by substituting the radius of QDs (*x* = 5 nm) into Eq. (3). The ${V}_{{FB}{\_}{TFB}}$, as determined by measuring the open-voltage ${V}_{{oc}}$ (Supplementary Fig. 6) or the electroabsorbtion spectra of the devices^22^, is slightly higher than the RT turn-on voltage of 1.6 V, indicating that the ${\phi }_{{h\_}{\min }}$ is smaller than 0.4 eV, which is reasonable and is in consistent with reported values ^1,3–5,11,19,23^.

(4) ${V}_{{FB\_TFB}} \, < \, V\le {hv}/e$ (Fig. 2e). As the applied voltage is larger than ${V}_{{FB\_TFB}}$, all depletion layers are vanished, and thus the applied voltage is mainly dropped across all layers. In this situation, the electric field in all layers turn positive, and thus the holes can be accelerated towards the QDs. The injection barrier is fixed at ${\phi }_{{h\_}{\min }}$. Now, the holes can gain their ${E}_{K}$ from both electric field and thermal energy, and thus hole injection is co-governed by the thermal-assisted and the field-assisted thermionic-emission mechanisms.

(5) $V \, > \, {hv}/e$ (Fig. 2f). When the applied voltage is larger than the photon voltage, a strong positive electric field is present in all layers. Most holes can gain their ${E}_{K}$ from the positive electric field and thus hole injection into QDs is mainly dominated by the field-assisted thermionic-emission mechanism. Very strong EL can be observed due to efficient charge injection.

From above analysis, we conclude that hole injection at sub-bandgap applied voltage, is mainly enabled by the thermal-assisted thermionic-emission mechanism, in which the holes can gain their ${E}_{K}$ from the thermal energy, consequently allowing them to overcome a barrier up to 0.8 eV at a applied voltage of 1.2 V and leading to the generation of 2.0 eV up-conversion photons.

### Thermal-assisted hole-injection

**Fig. 3:**
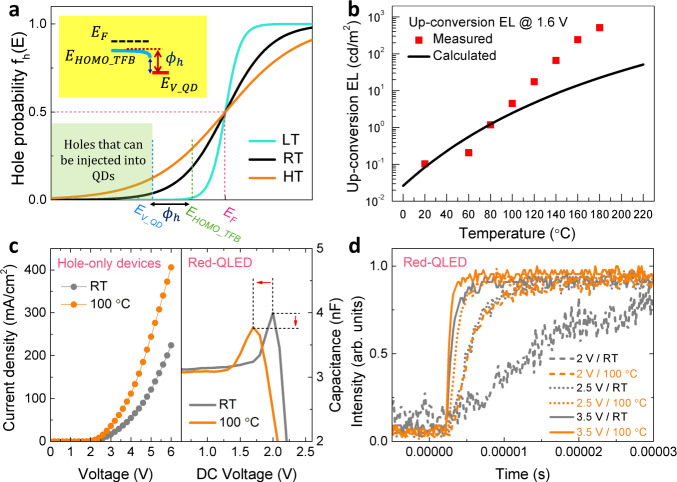
Thermal-assisted hole-injection. **a** The probability of finding the holes at different energy: when the temperature is elevated, the probability that the holes can be injected into QDs is remarkably increased. **b** Up-conversion EL at 1.6 V under different temperatures: the calculated results agree fairly well with the measured one when the temperature is lower than 80 °C. At high temperature, the discrepancy is caused by the thermal excitation that increases the hole concentration of TFB. **c** The current density (J)-V characteristics of hole-only devices and capacitance-V characteristics of the red QLEDs at RT and 100 °C: at elevated temperature, the hole current is substantially enhanced, which thus reduces the peak capacitance of the devices. **d** The transient EL of red-QLEDs: at elevated temperature, the devices are turned-on more rapidly due to enhanced hole injection.

A question remains unresolved is whether the thermal energy *kT* is sufficiently high enough to assist the holes overcoming the ${\phi }_{h}$. The *kT* at RT is only 0.026 eV, which seems too low to support the holes to overcome a barrier of 0.4 eV. However, it should be noted that the *kT* is a statistical average energy of many particles. Considering the thermal fluctuation, there has a probability that the energy of a specific particle is significantly higher than the average value, as described by the Boltzmann distribution law^26^. For the holes, the probability of finding them at a certain energy *E* is defined by a more rigorous Fermi-Dirac distribution law ^25^:

6 $${f}_{h}\left(E\right)=\frac{1}{1+{\exp }\left(\frac{{E}_{F}-E}{kT}\right)}$$

where ${E}_{F}$ is the Fermi level of TFB. The probability of finding the holes as a function of *E* is plotted in Fig. 3a. It is obvious that even at RT, there has a certain probability $\left[{f}_{h}\left({E}_{{V\_QD}}\right)\right]$ for the holes to overcome the barrier ${\phi }_{h}$, and inject into the valence band of QDs. When the temperature is elevated, the probability of hole injection into QDs is significantly increased. The amount of holes (per volume) that can be injected into QDs can be deduced by:

7 $$p={N}_{v}{e}^{-\frac{{E}_{F}-\left({E}_{{HOMO}{{{{{\rm{\_}}}}}}{\_}{TFB}}+{\phi }_{h}\right)}{{kT}}}={N}_{v}{e}^{-\frac{{E}_{F}-{E}_{{HOMO}{\_}{TFB}}}{{kT}}}{e}^{-\frac{{\Phi }_{h}}{{kT}}}={p}_{0}{e}^{-\frac{{\phi }_{h}}{{kT}}}\,$$

where ${N}_{v}$ is the effective density of state of TFB and ${p}_{0}$ is the hole concentration (number of holes cm^−3^) in TFB. Equation (7) implies that hole injection into QDs is affected by both temperature and injection barrier, which is the typical characteristics of the thermionic-emission mechanism. From above discussion, at RT, the holes can gain the ${E}_{K}$ from thermal energy and overcome a ${\phi }_{h}$ of 0.4 eV, that is:

8 $${E}_{K}=\frac{1}{2}{m}_{h}{{v}_{h}}^{2}={\phi }_{h}$$

where ${m}_{h}$ is the hole effective mass in TFB, and ${v}_{h}$ is the velocity of the holes that can overcome the barrier. Per unit area and per second, the amount of holes (${{{{{\rm{number}}}}}}\,{{{{{\rm{of}}}}}}\,{{{{{\rm{holes}}}}}}\,{{{{{{\rm{cm}}}}}}}^{-2}{{{{{{\rm{s}}}}}}}^{-1}$) that can be injected into QDs is:

9 $$N=p\times {v}_{h}={p}_{0}{e}^{-\frac{{\phi }_{h}}{{kT}}}\times \sqrt{\frac{2{\phi }_{h}}{{m}_{h}}}$$

Assuming all injected holes are used to generate the photons, then the photon flux per unit area (number of photons cm^−2^s^−1^) is:

10 $${{{\rm{\varphi }}}}=N$$

 The luminance (cd m^−2^) can then be calculated by:

11 $$L=\frac{\varphi \times {hv}\times \eta \times {\eta }_{{oc}}}{\varOmega }=\frac{{p}_{0}\times {hv}\times \eta \times {\eta }_{{oc}}}{\varOmega }\times {e}^{-\frac{{\phi }_{h}}{{kT}}}\times \sqrt{\frac{2{\phi }_{h}}{{m}_{h}}}$$

where ${{{{{\rm{\eta }}}}}}$ is the luminous efficacy (lm/W) of photopic vision, ${\eta }_{{oc}}$ is the outcoupling efficiency of the device and $\varOmega$ is the solid angle of the emission. By substituting ${p}_{0}={2.5\times 10}^{11}\,{{{{{\rm{c}}}}}}{{{{{{\rm{m}}}}}}}^{-3}$ ^27^, ${hv}=2{{{{{\rm{eV}}}}}}$, $\eta =260{{{{{\rm{lm}}}}}}/{{{{{\rm{W}}}}}}$,$\,{\eta }_{{oc}}=25 \%$, $\varOmega =3.14$, ${\phi }_{h}=0.4{{{{{\rm{eV}}}}}}$, ${m}_{h}=9.1\times {10}^{-31}\,{{{{{\rm{kg}}}}}}$ into Eq. (11), the luminance at a applied voltage of 1.6 V (corresponding to an injection barrier of 0.4 eV) can be calculated. As shown in Fig. 3b, the calculated luminance agrees fairly well with the measured value when the temperature is lower than 80 °C. At higher temperature, the measured value is higher than the calculated one, and this is because a constant ${p}_{0}$ is used to calculate the luminance, while at high temperature, the hole concentration of TFB can be significantly higher than ${p}_{0}$ due to thermal excitation, thus leading to the underestimation of the calculation. At low temperature, it is reasonable to use a constant ${p}_{0}\,$because the population of thermally generated holes is too low to affect the ${p}_{0}$. The above theoretical deduction and calculated results, which are based on classical carrier distribution and thermionic emission model, confirm that at RT, the holes can indeed overcome a barrier of 0.4 eV and inject into QDs via the thermal-assisted thermionic-emission mechanism, which consequently leads to the generation of detectable EL of 0.12 cd m^−2^ at a sub-bandgap voltage of 1.6 V.

The thermal-assisted hole injection can further be proved experimentally. Figure 3c shows the J–V of a hole-only-device under different temperatures. At elevated temperature, the hole current is substantially enhanced, and as a result, the amount of the accumulated electrons is effectively reduced by recombining with the injected holes. Consequently, the peak capacitance of the devices is reduced and shifted to the low voltage region, as demonstrated in Fig. 3c. Moreover, at elevated temperature, the device turns-on more rapidly, as disclosed by the transient EL (Fig. 3d), which also suggests that hole injection is substantially enhanced by increasing the temperature. The thermal energy, not only assists the holes to overcome the barrier, but also promotes the hopping transport of the holes^28^; both are contributed to an improved hole-injection.

**Fig. 4:**
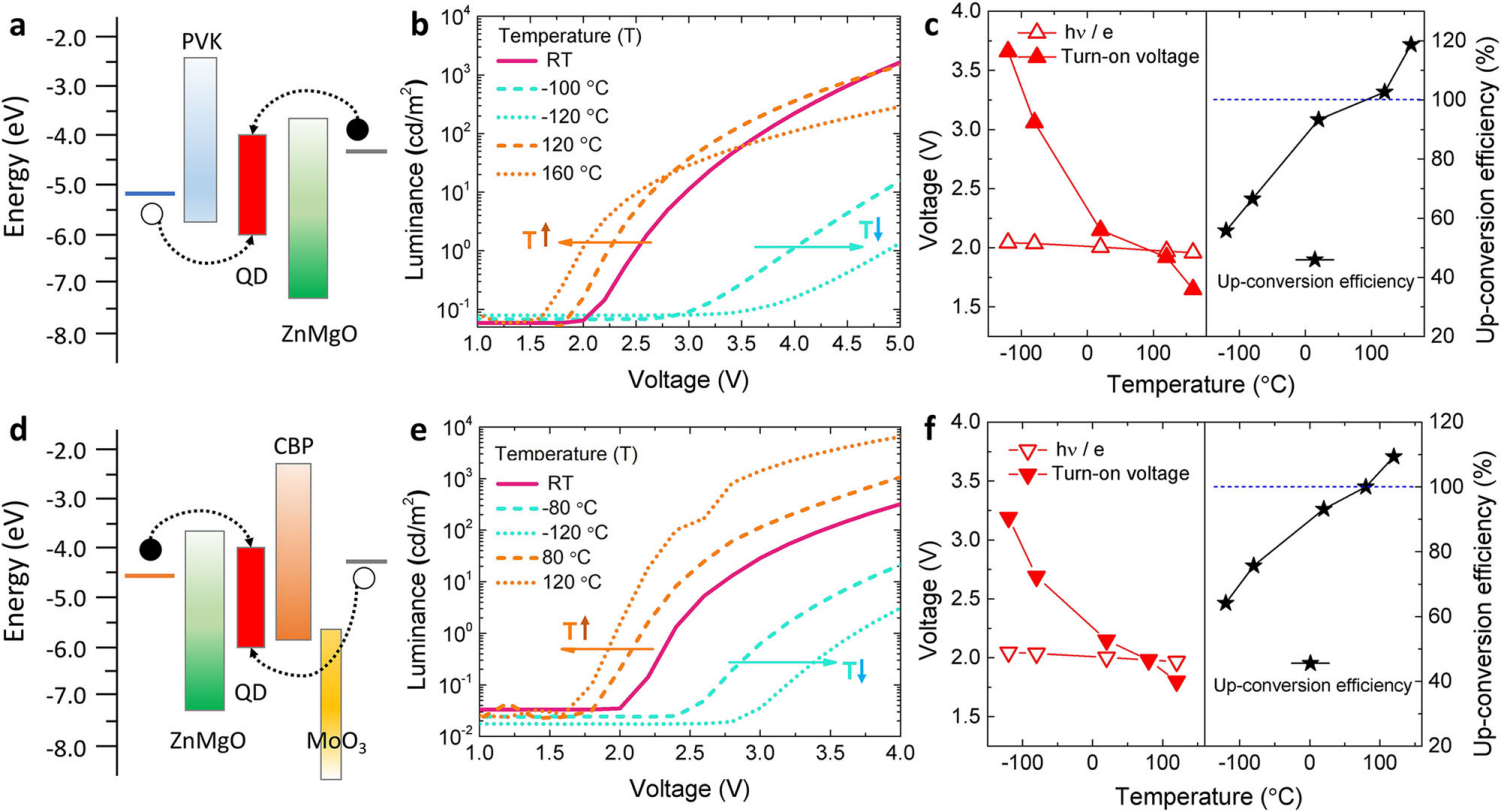
The up-conversion EL in different structured QLEDs. The energy levels diagrams of **a** regular red-QLEDs with PVK HTL and **d** inverted red-QLEDs with CBP HTL. (black/white circle represents electron/hole). The L–V characteristics of **b** PVK based- and **e** CBP based-QLEDs under different temperatures: the ${V}_{T}$ is remarkably reduced as the temperature is increased. Turn-on voltage ${V}_{T}$ (solid triangle), photon voltage ${V}_{{ph}}$ (open triangle) and the up-conversion efficien**c**ies (star) of **c** PVK based- and **f** CBP based-QLEDs at different temperatures: the up-conversion efficiency is gradually increased as the temperature is increased. When the temperature is higher than 100 °C, the up-conversion efficiency of both QLEDs can exceed 100%.

Finally, we show that the thermal-assisted hole-injection is a universal mechanism for the up-conversion EL observed in different structured and different colored QLEDs. The up-conversion EL has been commonly observed in regular structured CdSe-based or InP-based (Supplementary Fig. 7) QLEDs with TFB HTL and ZnO-based ETL. By replacing the TFB with PVK or CBP, it is difficult to observe the up-conversion EL at RT, because the hole injection, which is significantly affected by ${p}_{0}$ and ${\phi }_{h}$, in these devices are completely different to that in devices with TFB HTL. However, as shown in Fig. 4, when the temperature is varied, all devices exhibit the same temperature-dependent EL characteristics. Although these devices are built with different HTLs and different architectures, they all show the up-conversion EL at elevated temperature, which can be well explained by the universal thermal-assisted hole-injection mechanism.

## Discussion

In summary, we address a fundamental question of how the holes are injected into the QDs at sub-bandgap bias that results in the up-conversion EL. Based on the experimental results of temperature-dependent EL, we reveal that thermal energy plays an essential role in the sub-bandgap charge injection processes. Theoretical analysis discloses the detail of the hole injection processes under different applied bias. Our results show that at RT and at an applied voltage of 1.6 V, an electron with energy of 1.6 eV can be directly injected into QDs, while with the assistance of thermal energy, a hole can overcome a barrier of 0.4 eV and inject into QDs, thereby resulting in a 2 eV up-converted photon. The up-conversion gain can be higher than 167% by increasing the temperature. Further theoretical deduction and experimental results confirm that thermal-assisted hole-injection is the universal mechanism responsible for the up-conversion EL. Our findings uncover the charge injection process and unlock the up-conversion EL mechanism, which could encourage the development of up-conversion QLEDs with power conversion efficiency over 100%.

## Methods

### Materials

All materials are commercially available. CdSe-based colloidal red-/green-/blue-QDs were purchased from Suzhou Xingshuo Nanotech Co., Ltd. ZnMgO nanoparticles were purchased from Guangdong Poly OptoElectronics Co., Ltd. Poly[(9,9-dioctylfluorenyl-2,7-diyl)-co-(4,4’-(N-(pbutylphenyl))diphenylamine)] (TFB) were purchased from American Dye Source, Inc. Poly(3,4-ethylenedioxythiophene)-poly(styrenesulfonate) (PEDOT:PSS), Poly(9-vinlycarbazole) (PVK), and 4,4’-Bis(9-carbazolyl)−1,1’-biphenyl (CBP) were purchased from Luminescence Technology Corp. Molybdenum trioxide (MoO_3_), chlorobenzene, and octane were purchased from Aladdin Industrial Corp. Absolute ethanol were purchased from ShangHai LingFeng Chemical Reagent Co., Ltd. ITO glass (20 Ω/sq) were purchased from Wuhu Jinghui Electronic Technology Co., Ltd.

### Device fabrication

QLEDs with regular structures of glass/ITO/PEDOT:PSS (45 nm)/TFB (40 nm)/QDs (~15 nm)/ZnMgO (40 nm)/Al (100 nm) and glass/ITO/PEDOT:PSS (45 nm)/PVK (40 nm)/QDs (~15 nm)/ZnMgO (40 nm)/Al (100 nm), and inverted QLEDs with a structure of glass/ITO/ZnMgO (45 nm)/QDs (~15 nm)/CBP (40 nm)/MoO_3_ (8 nm)/Al (100 nm) were fabricated. ITO/Al, PEDOT:PSS/MoO_3_, TFB/PVK/CBP, QDs, and ZnMgO nanoparticles work as electrode, hole injection layer (HIL), hole transport layer (HTL), light emission layer (EML), and electron transport layer (ETL), respectively. The hole-only devices with a structure of glass/ITO/PEDOT:PSS (45 nm)/TFB (40 nm)/QDs (~15 nm)/Al (100 nm) were fabricated to investigate the hole injection under varied temperatures.

For the regular red QLEDs, the cleaned ITO glass substrates were treated with O_2_ plasma for 6 min firstly. Next, the HILs were formed by spin-casting PEDOT:PSS solution at 3000 rpm and baked at 130 °C for 20 min in the atmosphere. Then, the PEDOT:PSS-coated samples were transferred into a nitrogen-filled glove box to prepare the subsequent functional layers. The TFB (8 mg mL^−1^ in chlorobenzene) HTLs were spin-coated on the top of PEDOT:PSS at 3000 rpm for 45 s and baked at 130 °C for 20 min. Subsequently, the EMLs were deposited by spin-casting the red QDs solution (15 mg mL^−1^ in octane) at 3000 rpm and baked at 100 °C for 5 min. Afterward, ZnMgO ETLs (nanoparticles, 20 mg mL^−1^ in ethanol) were spin-coated on QDs films at 2500 rpm and baked at 100 °C for 10 min. Finally, the coated samples were transferred to a high-vacuum evaporation chamber to deposit a 100 nm Al cathode with an evaporation rate of 5 Å s^−1^ at a base pressure of 4 × 10^−4^ Pa. For the PVK HTLs, a 10 mg mL^−1^ PVK solution in chlorobenzene was used. The regular green and blue QLEDs were fabricated by the same procedure by using a 10 mg mL^−1^ green QDs solution (in octane), and a 10 mg mL^−1^ blue QDs solution (in octane), respectively. For inverted red QLEDs, ZnMgO ETLs and QDs EMLs were directly deposited on ITO glass. Then, the samples were transferred to a high-vacuum evaporation chamber. At a base pressure of 4 × 10^−4^ Pa, CBP HTLs and MoO_3_ HILs were deposited layer by layer with rates of 1.5 and 0.2 Å s^−1^, respectively. Al anodes were deposited by the same process as conventional ones. In the end, the QLEDs were encapsulated with UV-resin and cover glass.

### Characterizations

The thicknesses of the functional layers were measured using a Bruker DektakXT stylus profiler. The evaporation rates and the thicknesses of CBP, MoO_3_, and Al electrode, were in situ monitored by a quartz crystal microbalance. EL spectra of QLEDs were measured by a fiber-optic spectrometer (USB 2000, Ocean Optics) in the normal direction, and the J–V–L characteristics of QLEDs were assessed by using a dual-channel Keithley 2614B programmable source meter with a PIN-25D calibrated silicon photodiode (PD) under ambient conditions. The C-V test was conducted by using an HP4284A LCR analyzer, and the frequency and amplitude of the AC signal are 1000 Hz and 0.05 V, respectively. The transient EL measurement was accomplished by using a signal generator (JunCe Instruments, JDS6600) to generate a square-wave signal (100k Hz), a PD (THORLABS, APD120A2/M) to receive lights generated by QLEDs, and a dual-channel oscilloscope (Tektronix, TBS1102) to receive the square-wave signal and the light-emitting response signal of the device.

To carry out the temperature-dependent experiments, a custom designed temperature-controllable probe station was used. Liquid nitrogen is used to control the temperature with a minimum accuracy of 0.1 °C. Temperature-dependent EL spectra/J–V–L characteristics/transient EL/C-V were all implemented using this probe station.

### Reporting summary

Further information on research design is available in the Nature Research Reporting Summary linked to this article.

## Supplementary information

Supplementary Information

Description of Additional Supplementary Files

Supplementary Movie 1

Reporting Summary

## Data Availability

The data that support the findings of this study are available from the corresponding author upon reasonable request.
