# Supplementary material for: Thermal assisted up-conversion electroluminescence in quantum dot light emitting diodes
Source: Nat Commun. 2022 Jan 18;13:369. doi: 10.1038/s41467-022-28037-w (PMC8766545; doi:10.1038/s41467-022-28037-w)
Supplement: Supplementary file 1 — Supplementary Information [file 41467_2022_28037_MOESM1_ESM.pdf]

Supplementary Information

Thermal-assisted up-conversion electroluminescence in quantum-dot light-emitting diodes

Qiang Su[1], Shuming Chen[1, 2]*
[1] Department of Electrical and Electronic Engineering, Southern University of Science and Technology, Shenzhen, 518055, P. R. China
[2] Key Laboratory of Energy Conversion and Storage Technologies (Southern University of Science and Technology), Ministry of Education, Shenzhen, 518055, P. R. China
* Corresponding author: Shuming Chen (chen.sm@sustech.edu.cn)

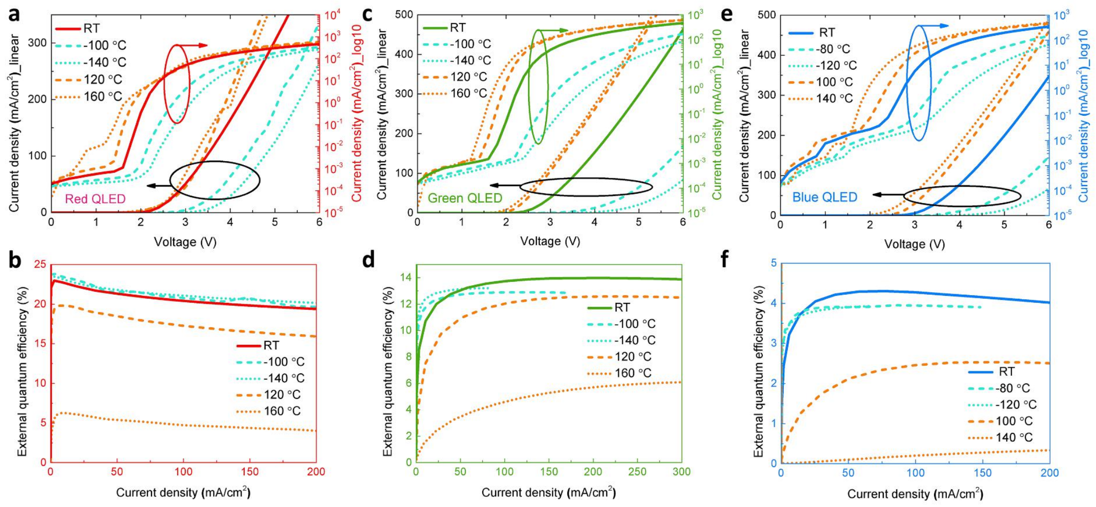

Supplemnetary Figure 1. The influence of thermal energy on QLEDs performance. The current density-voltage (J-V) and external quantum efficiency (EQE) -J characteristics of **a b** R-, **c d** G-, and **e f** B-QLED under different temperatures. The current density (linear and log10 represent linear and logarithmic coordinates, respectively) of QLEDs is remarkably increased (orange lines) at elevated temperatures due to the enhanced charge injection. Although the injection is promoted and the brightness is enhanced, the EQE is reduced due to the damage of QDs and the quenching of excitons at elevated temperatures.

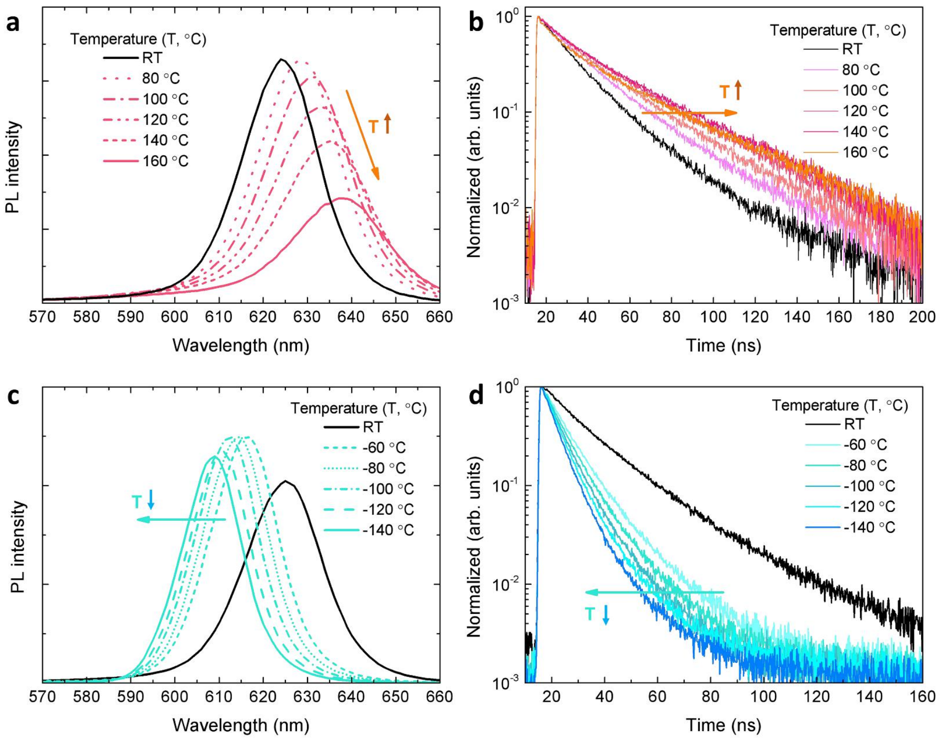

Supplemnetary Figure 2. Temperature-dependent steady PL spectra and time-resolved PL of the sample with the structure of glass/TFB/QDs. PL spectra showed a red shift **a** at high temperatures, and a blue shift **c** at low temperatures. The change of E_g with temperature can be explained by Varshni equation: $E_g = E_{g0} - \frac{\alpha T^2}{(T+\beta)}$, where E_{g0} is the bandgap at 0 K, α is the temperature factor, and β is the parameter related to the Debye temperature of the material. **a b** At elevated temperatures, thermal-assisted recombination occurs, which increases the quenching possibility and thereby reduces the PL intensity of QDs. At elevated temperatures, the electrons are delocalized to the surface traps first and then relax back to the core and recombine with the confined holes, thereby leading to the prolonged exciton lifetime. **c d** On the contrary, at reduced temperatures, thermal-assisted recombination is suppressed, and as a result, the PL intensity is increased and the exciton lifetime is shortened.

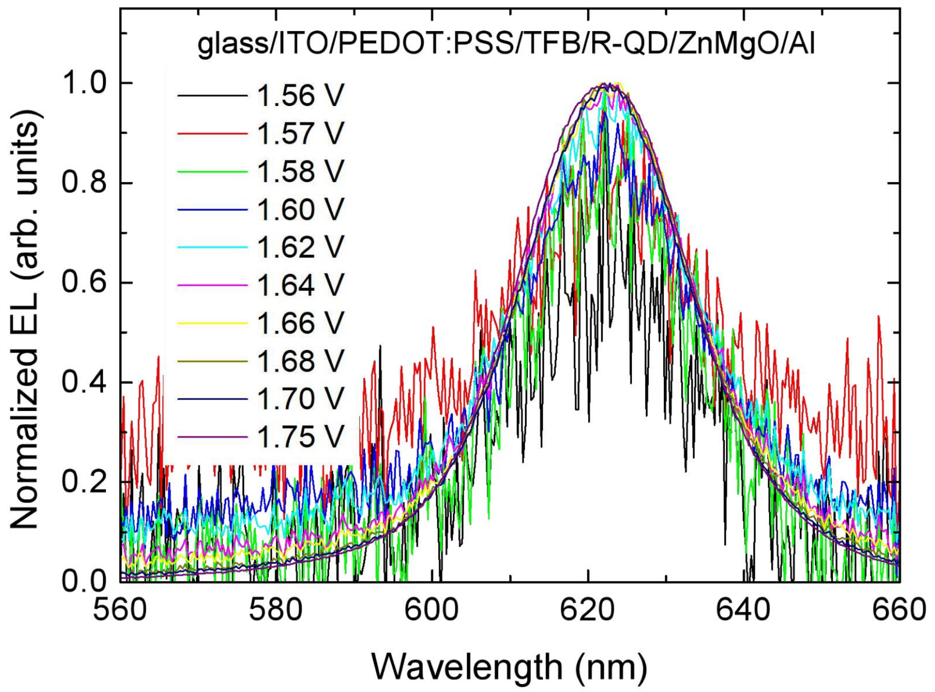

Supplemnetary Figure 3. EL spectra of red-QLEDs at the voltage near turn-on voltage. Even at a sub-bandgap applied voltage, the EL spectra are identical to those at high voltage, indicating that the emission is originated from the QDs.

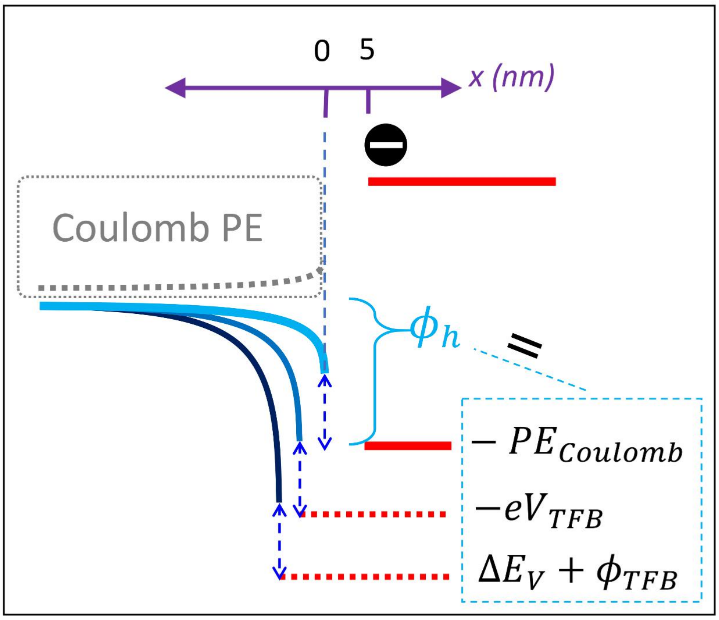

Supplemnetary Figure 4. The heterojunction barrier between TFB and QDs. The hole injection barrier is determined by $\phi_h = \Delta E_V + \phi_{TFB} - eV_{TFB} - PE_{Coulomb}$. Where ΔE_V is the heterojunction barrier between TFB and QDs; ϕ_{TFB} is the build-in surface potentials at the surface of TFB due to the presence of surface depletion layer; V_{TFB} is the effective applied voltage that is dropped across the depletion layer of TFB, and $PE_{Coulomb}$ is the potential energy of holes caused by Coulombic interaction. When the applied voltage is increased to V_{FB_TFB}, the accumulated electrons in QDs tend to attract the holes in TFB via the Coulombic interaction. $PE_{Coulomb}$ is estimated to be ~0.1 eV by substituting the radius of QDs (*x*=5 nm) into:

$$PE_{Coulomb} = \frac{e^2}{4\pi\varepsilon_0\varepsilon_r x}$$

The gray dotted line represents the hole potential energy induced by Coulombic interaction.

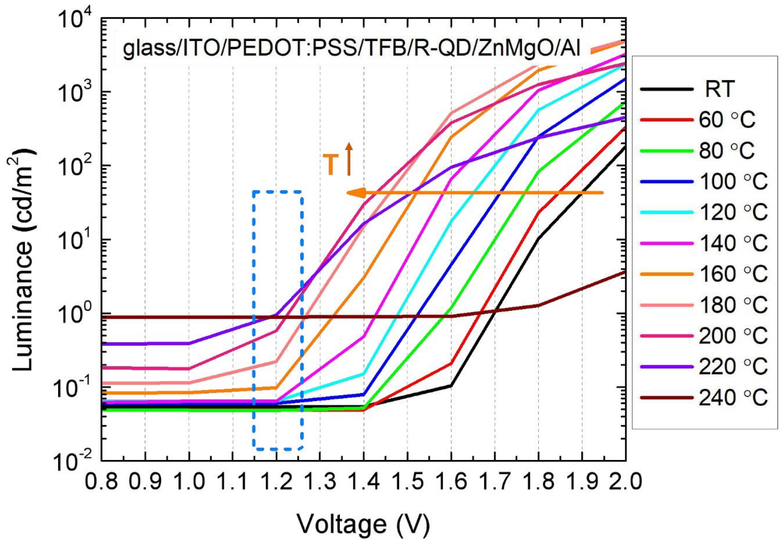

Supplemnetary Figure 5. The minimum voltage to initiate the EL. The V_{FB_QD} is the minimum voltage to initiate the EL, and thus by measuring the lowest V_T, the V_{FB_QD} can be accessed. When the temperature is increased from 160 to 240 °C, the V_{T_HT} (turn-on voltage at HT) cannot be further reduced and is fixed at ~1.2 V, which represents the lowest V_T and therefore marks the value of V_{FB_QD}.

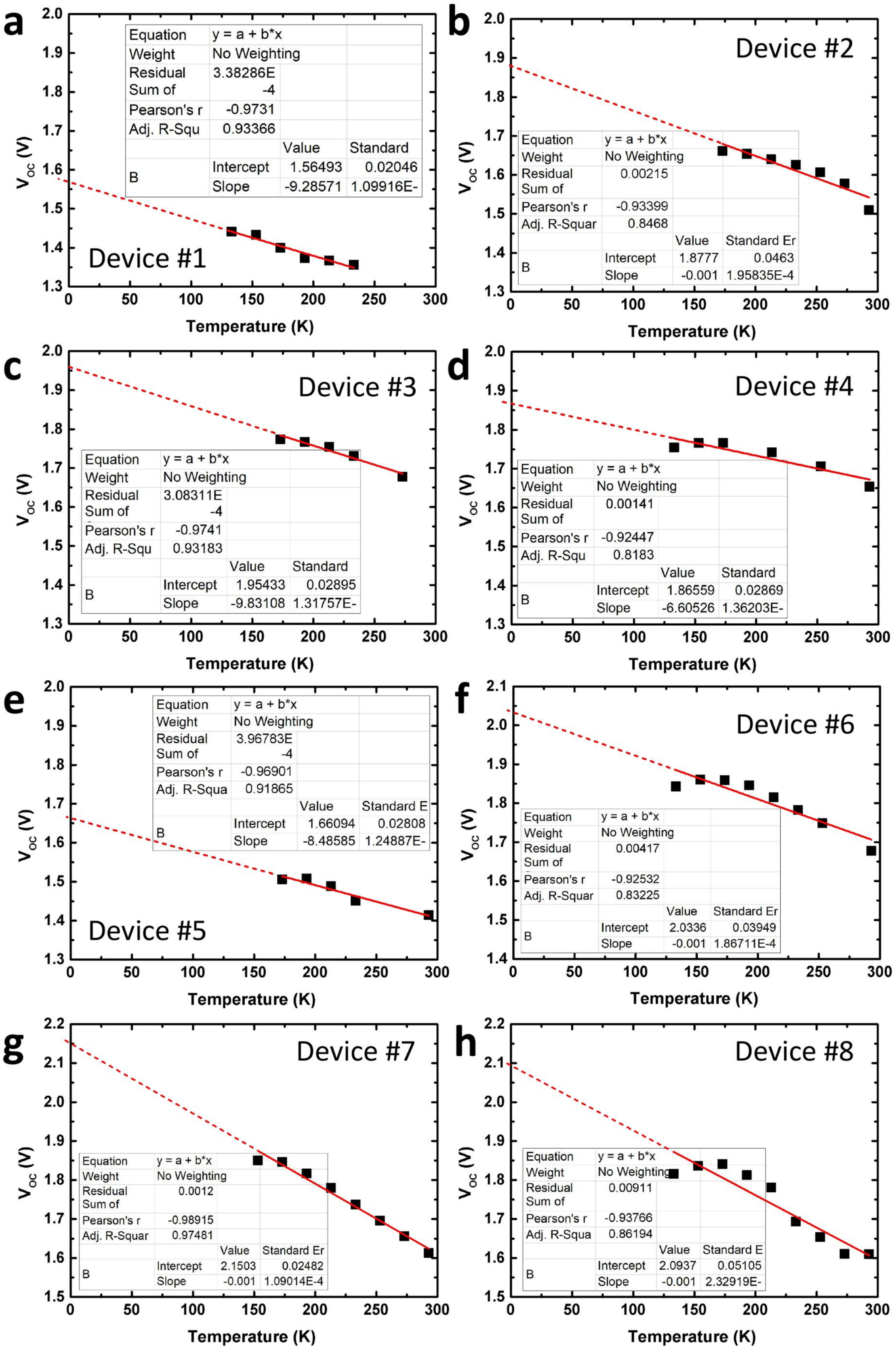

Supplemnetary Figure 6. The QLEDs worked in the solar cell mode. a-h The open-circuit voltages (V_{OC}) of eight red-QLEDs under different temperatures when working in the solar cell mode. Placing the QLEDs under the irradiation of a AM1.5

sunlight simulator, it can work as a solar cell and thus outputted photogenerated voltage. For a typical solar cell based on heterojunction, the V_{OC} mainly depends on the energy difference between the conduction band of the N-side and the valence band of the P-side, and thus can be used to evaluate the V_{FB_TFB}.

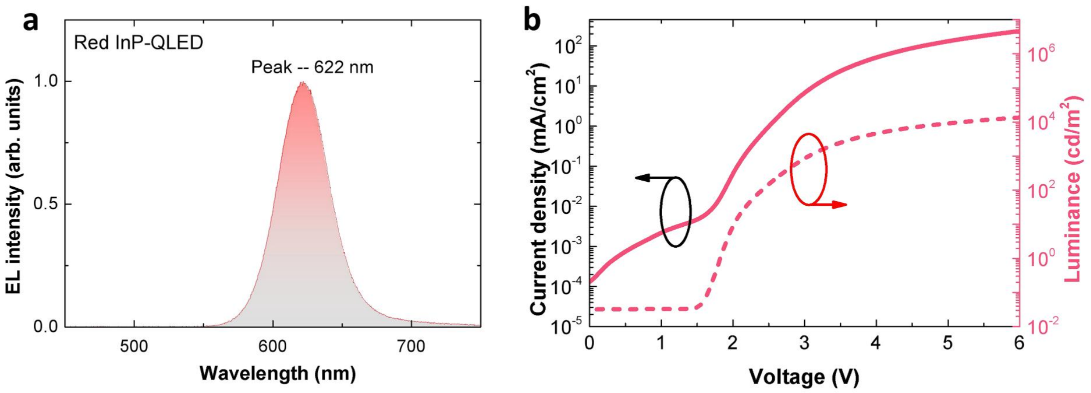

Supplemnetary Figure 7. The up-conversion EL phenomenon at room temperature in InP-based QLEDs with a structure of glass/ITO/PEDOT:PSS/TFB/red InP QDs/ZnMgO/Al. a The EL spectrum and **b** the J-V and luminance-V (L-V) characteristics of the red InP-QLED. The turn-on voltage to induce a luminance of 0.1 cd m^{-2} of red InP QLEDs (622 nm) is about 1.6 V (dashed line in **b**), which are remarkably lower than their corresponding photon voltages ($h\nu/e$) of 2.0 V. Both InP-based and Cd-based QLEDs have the similar charge transport layer, and thus the mechanism of up-conversion EL should be the same.
