# Supplementary material for: Thermal assisted up-conversion electroluminescence in quantum dot light emitting diodes
Source: Nat Commun. 2022 Jan 18;13:369. doi: 10.1038/s41467-022-28037-w (PMC8766545; doi:10.1038/s41467-022-28037-w)
Supplement: Supplementary file 2 — Description of Additional Supplementary Files [file 41467_2022_28037_MOESM2_ESM.pdf]

Description of Additional Supplementary Files

File Name: Supplementary Movie 1

Description: Thermal assisted up-conversion EL in QLEDs: at a subbandgap bias of 1.6 V, the up-conversion EL is switched on at RT and is gradually enhanced when the temperature is further elevated. At a temperature of 200 ℃, an electron with energy of 1.2 eV can be up-converted to a 2.0 eV photon.
